# Clinical and economic efficiency to reduce dose etanercept and adalimumab in patients with rheumatoid arthritis

**DOI:** 10.1186/1479-5876-10-S3-P51

**Published:** 2012-11-28

**Authors:** Rosalía Martínez-Pérez, Sergio Rodríguez-Montero, Alejandro Muñoz, Julia Uceda, Mario León, Francisco Gallo, Maria Luisa Velloso, José Luis Marenco

**Affiliations:** 1 Rheumatology Unit, Valme University Hospital, Sevilla, Spain

## Objective

To assess the clinical and economic efficiency as well as functional capacity in patients with rheumatoid artritis treated with biologic after dose reduction.

## Methods

A retrospective study on a cohort of 13 patients diagnosed rheumatoid artritis (RA) treated with anti-TNF (10 patients with etanercept, and 3 with adalimumab) and clinical remission (DAS28 < 2.6) in the last 6 months, in which reducing the dosing. This reduction was about 30% in both groups (etanercept passed from 25 mg twice a week to 25 mg every 5 days. In the case of Adalimumab it went from 40 mg to 40 mg every 2 weeks every 20 days.

Clinical activity was evaluated by clinical activity index (DAS28), for functional capacity was used HAQ (Health Assessment Questionnaire). Others variables studied was visual analog scale (VAS). Analyzing before starting dose reduction, at 3 and 6 months.

## Results

The study includes 13 patients, 12 female, with RF in 90% and erosions in 40%, treated with etanercept 10 and adalimumab 3. More of them (85%) had recieved at least 2 FAMES before biological. Dose reduction in the etanercept group was 2 weekly injections to one injection every 5 days. In the case of adalimumab the pattern used was an injection every 20 days.

**Table 1:** 

	BASELINE	3 months (p baseline-3m)	6 months (p 3m-6m)	INFERENCE
VAS (mm)				
-ETN	21.00±16.63	26.50±18.56 (p = 0.048)	23.89±17.28 (p = 0.22)	p = 0.89
-ADA	30.00±20.00	38.67±18.03 (p = 0.49)	20.00±19.36 (p = 0.54)	p = 0.36

HAQ				
-ETN	0.48±0.50	0.61±0.45 (p = 0.33)	0.49±0.40 (p = 0.34)	p = 0.67
-ADA	1.33±0.21	1.66±0.28 (p = 0.19)	1.93±0.26 (p = 0.25)	p = 0.003

DAS28				
-ETN	2.15±0.45	2.74±0.85 (p = 0.056)	2.41±0.77 (p = 0.005)	p = 0.20
-ADA	2.13±0.47	3.85±1.07 (p = 0.97)	2.98±1.03 (p = 0.005)	p = 0.005

60% (6 / 10) of patients with etanercept was maintained with a reduced schedule after 6 months, and 33% (1/3) of patients treated with adalimumab. During the study period was generated savings of EUR 17,768.90 for etanercept and adalimumab 5786.91 euros related to the dose reduction.

## Conclusions

The reduction of etanercept and adalimumab doses in patients with rheumatoid arthritis in clinical remission (DAS28 < 2.6), It seems to be an effective strategy given that in the patients in this study were able to maintain the good clinical (DAS 28 and VAS) in 60% of patients treated with etanercept and 33% with adalimumab. Not so the HAQ in which if there are significant differences that support that after the patient expresses reduce doses higher score on the test. Although the results 6 of 13 patients (46.2%) return to your regular schedule by mutual self-referred as subjectively feel worse and increase in seizures of exacerbation.

In the light of the results of this study the dosage reduction can be an efficient alternative at the time of treating patients with RA into remission.

